# Normalization for triple-target microarray experiments

**DOI:** 10.1186/1471-2105-9-216

**Published:** 2008-04-28

**Authors:** Marie-Laure Martin-Magniette, Julie Aubert, Avner Bar-Hen, Samira Elftieh, Frederic Magniette, Jean-Pierre Renou, Jean-Jacques Daudin

**Affiliations:** 1 UMR AgroParisTech-INRA MIA 518, 75231 Paris Cedex05, France; 2 UMR INRA 1165-CNRS 8114-UEVE URGV, 91057 Evry Cedex, France; 3 Unité MOY300, Délégation CNRS île de France Est, 94532 Thiais Cedex, France; 4 Universite Paris Descartes, MAP 5, Paris cedex 06, France

## Abstract

**Background:**

Most microarray studies are made using labelling with one or two dyes which allows the hybridization of one or two samples on the same slide. In such experiments, the most frequently used dyes are *Cy*3 and *Cy*5. Recent improvements in the technology (dye-labelling, scanner and, image analysis) allow hybridization up to four samples simultaneously. The two additional dyes are *Alexa*488 and *Alexa*494. The triple-target or four-target technology is very promising, since it allows more flexibility in the design of experiments, an increase in the statistical power when comparing gene expressions induced by different conditions and a scaled down number of slides. However, there have been few methods proposed for statistical analysis of such data. Moreover the lowess correction of the global dye effect is available for only two-color experiments, and even if its application can be derived, it does not allow simultaneous correction of the raw data.

**Results:**

We propose a two-step normalization procedure for triple-target experiments. First the dye bleeding is evaluated and corrected if necessary. Then the signal in each channel is normalized using a generalized lowess procedure to correct a global dye bias. The normalization procedure is validated using triple-self experiments and by comparing the results of triple-target and two-color experiments. Although the focus is on triple-target microarrays, the proposed method can be used to normalize *p *differently labelled targets co-hybridized on a same array, for any value of *p *greater than 2.

**Conclusion:**

The proposed normalization procedure is effective: the technical biases are reduced, the number of false positives is under control in the analysis of differentially expressed genes, and the triple-target experiments are more powerful than the corresponding two-color experiments. There is room for improving the microarray experiments by simultaneously hybridizing more than two samples.

## Background

DNA microarray technology is a high throughput technique by which the expression of the whole genome is studied in a single experiment. In dual label experiments the fluorescent dyes *Cy*3 and *Cy*5 are used to label the two RNA samples co-hybridized on a same array. Recently two more dyes have been proposed (*Alexa *488 and *Alexa *594) allowing the simultaneous hybridization of three or four samples. Forster et al. [2] have evaluated triple-target microarray by comparing results of single-target, dual-target and triple-target microarrays. They have concluded that the use of triple-target microarray is valid from an experimental point of view. One year later, Staal et al. [7] have investigated the four-target microarray experiments. Their approach differs from that of [2], but their conclusions are in fair agreement. Their study has shown that *Alexa *594 is best suited as a third dye and that *Alexa *488 can be applied as a fourth dye on some microarray types. These extensions of the microarray technology are promising because they increase throughput, minimize costs and allow more powerful design of experiments. Despite these advantages, triple-target microarrays are only sparsely used [6]. The lack of guidelines for designing these experiments and for normalizing more than two-color microarray data may be an explanation. Recently Woo et al. [8] have proposed experimental designs for three and four-color gene expression microarrays. According to the previous work of [2], the lowess procedure [9] used to normalize data from two-color microarray is still applicable but it normalizes data sequentially because the MA-plot or the lowess correction is defined only for two dyes. Consequently, application of such a normalization method does not globally correct the dye bias due to the three dyes. Moreover the introduction of a third dye induces signal "bleeding". Forster et al. [2] have concluded that "it was considered as negligible between *Cy*3 and *Cy*5 signals, but seems to be important between *Alexa*594 and *Cy*3 signals," therefore signal cross-talk cannot be neglected.

We propose in this paper a normalization method for triple-target microarray experiments. First we quantify and correct the signal bleeding. Then we correct the global dye bias using a generalized lowess procedure. Triple-target experiments with *Arabidopsis thaliana *microarrays are used to check if the proposed normalization is effective for correcting the dye bias. Moreover the comparison of the statistical power of the triple-target experiment versus the usual two-color experiment is performed. All programs and data produced for this project are available under request.

## Results and Discussion

### Bleeding

Using the vocabulary of [2], we call a channel, a *blank *channel when no material is hybridized for the associated dye. In theory, this blank channel should produce no signal values, and deviations from this show a bleeding phenomena from one dye-label to another. Signal bleeding from one dye-labelled sample to another is a potential source of bias. Indeed, bleeding artificially increases the signal in other channels of the same spot when the signal is high in one channel. Assume that a gene is highly expressed in condition A and weakly expressed in condition B. The difference between the two conditions is decreased by the bleeding. Therefore bleeding may induce a lowering in the statistical power for detecting differentially expressed genes. Another possibility is that the bleeding effect induces a difference between two channels for the same gene: assume that a gene is highly expressed in condition A and equally expressed in conditions B and C; if the bleeding between the channel corresponding to condition A and the channel corresponding to condition B is higher than the bleeding between A and C, then a difference between signals B and C will appear, which is a technical artifact.

In order to investigate bleeding, we have made a "single target hybridization microarray experiment" where only one dye-labelled sample is hybridized (see the dataset URGV1 in the Methods Section). We also analyzed the single target hybridization data set from Forster [2].

#### Experimental indications of the existence of bleeding

**Figure 1:**
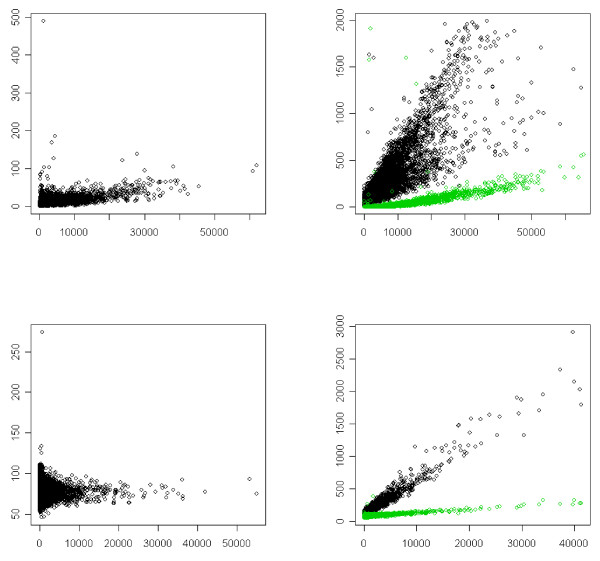
**Bleeding**. First row: Forster data, last row: URGV1 data. In the first column the hybridized dye is *Cy*3 and the *empty *dye is *Cy*5, in the second column the hybridized dye is *Alexa*594 and the *empty *dyes are *Cy*3 (black) and *Cy*5(green). *x-*axis: signal along the hybridized channel, y-axis: signal along the *empty *channel(s).

Figure 1 gives some plots between the hybridized and blank channels for the Forster and URGV1 datasets. These plots illustrate the bleeding. This bias depends on the channel: the bleeding bias *Cy*3 → *Cy*5 is negligible but the bleeding bias *Alexa*594 → (*Cy*5, *Cy*3) exists. The plots from the Forster single target hybridization experiment and URGV1 datasets present the same patterns with a greater variance for the first set. As the bleeding effect seems to apply on a linear scale, we consider only raw (and not log-transformed) data in this section. Table 1 which contains the Spearman correlation coefficients between the hybridized and the blank channels for the two datasets shows that the bleeding effect exists. The correlations between *Cy*5 and *Cy*3 are low, but the dye *Alexa*594 emits and receives significant cross-talk from the other two dyes.

**Table 1:** Bleeding: correlations between hybridized and empty channels. Mean (standard error of the mean) Spearman correlations between hybridized and empty channels.

Data	Cy5 → Cy3	Cy5 → Alexa	Cy3 → Cy5	Cy3 → Alexa	Alexa → Cy5	Alexa → Cy3
Forster	0.29 (0.06)	0.75 (0.002)	0.39 (0.06)	0.84 (0.11)	0.82 (0.02)	0.83 (0.02)
URGV1	0.13 (0.06)	0.58 (0.04)	0.02 (0.03)	0.47 (0.05)	0.26 (0.03)	0.61 (0.04)

Since cross-talks exist, we quantify then by using linear regression models. For example, when the sample is hybridized with *Alexa*594 and *Cy*5 and *Cy*3 are the blank channels, the following models are used: *G*_*i *_= *α*_1 _+ *β*_1_*Y*_*i *_+ *ε*_*i *_and *R*_*i *_= *α*_2 _+ *β2Y*_*i *_+ ${\varepsilon^{\prime }}_{i}$, where *G*, *Y *and *R *stand respectively for green, yellow and red signals and *i *denotes the spot index. Similar models are used with swapped dyes. Estimation is performed using a robust method (R-function *rlm*, [3]) to decrease the effect of outliers. Table 2 contains the estimated parameters, which are low. This shows that the impact of bleeding on the signal is low. The greater coefficient is between *Cy*3 and *Alexa*594 (0.07). The weakness of the quantitative influence of bleeding is confirmed by the values of the standard error of the signal in the different channels: the values for the empty channels are between 6 and 200 times lower than the corresponding values for hybridized targets (Table 3). These conclusions are made for only three dyes and two experimental platforms. It is possible that other dyes or other laser technologies induce a greater bleeding bias.

**Table 2:** Bleeding: regression coefficient between hybridized and empty channels. Mean (se) of the regression coefficient (x1000) between hybridized and empty channels (robust regression method).

Data	Cy5 → Cy3	Cy5 → Alexa	Cy3 → Cy5	Cy3 → Alexa	Alexa → Cy5	Alexa → Cy3
Forster	1 (1)	6 (3)	0.5 (0.5)	26 (14)	2.5 (0.3)	27 (5)
URGV1	1.0 (0.1)	52 (5)	0.0 (0)	26 (2)	5 (0.4)	70(15)

**Table 3:** Bleeding: Standard deviation of the signal in each channel. The hybridized target signal values are in bold.

Forster experiment			
Slide	*Alexa*	*Cy*3	*Cy*5

6s	**8043**	277	193
11s	**6845**	251	191
16s	**6704**	368	245
10s	1132	1210	**6802**
17s	585	819	**6936**
18s	264	**7240**	219
23s	1033	**4188**	939
			

URGV experiment			

Slide	*Alexa*	*Cy*3	*Cy*5
3	**1249**	73	10
6	**1124**	96	10
2	65	16	**1323**
5	78	41	**1346**
1	48	**1313**	7
4	45	**1739**	7

#### Correction of bleeding

When there is a high level of bleeding it is necessary to correct it. A procedure is described in the Methods section in order to fulfill this objective. It necessitates a preliminary experiment with three single-target slides. We have used the bleeding correction for the URGV dataset in the following studies. However the results obtained are very similar with and without bleeding correction, because the importance of bleeding is not sizeable, so the data have not been corrected for bleeding in the following studies.

Note that the bleeding bias is cut down by a complete or partially dye-balanced experimental design, because the measure of the expression difference between two conditions is the mean of the individual measures of this difference taken on each slide. For example, if only one difference is distorted by the bleeding bias, its influence on the mean difference of expression is divided by the number of terms in the mean, which is equal to the number of slides containing the two conditions.

### Normalization of the dye bias

Dye bias is a well characterized technical bias occuring in two-color microarray. It is mainly due to an incorporation difference between the two dyes. We refer to [4,9] for details on this bias and also to [5] for the gene-specific dye bias. This bias is the most important technical bias and must be corrected before any transcriptome data analysis. The most used method is the lowess correction proposed by [9]. In triple-target microarray, this bias also exists and must be corrected. Unfortunately the lowess correction is defined only for two dyes. Thus for the triple-target microarrays, [2] used the lowess correction for three dye-label combinations per array: *Cy*5/*Cy*3, *Cy*5/*Alexa*594 and *Cy*3/*Alexa*594. However, this procedure does not allow a global correction of the dye bias. In this paper we propose a new method generalizing the lowess correction to correct the dye bias in one step.

Let *i *= 1, ⋯, *n *be the gene index (*i *is actually the spot index, but in the following we call it loosely the gene index), *j *= 1, ⋯, *p *the channel index and, *y*_*ij *_the log_2 _transformed intensity measure of gene *i *along the channel *j*. Let ${\bar{Y}}_{i}=\frac{1}{p}\sum_{j}Y_{ij}$, be the *mean channel *raw data for gene *i *on the log scale, and $D_{ij}=Y_{ij}-{\bar{Y}}_{i}$, the difference between channel *j *and the mean channel for gene *i*. We generalize the lowess method by modelling *D*_*ij *_as follows

$$D_{ij}=f_{j}({\bar{Y}}_{i})+E_{ij}$$

and by estimating *f*_*j *_via a lowess. The value of the channel *j *after normalization of intensity dye-bias is defined by:

(1) $${\tilde{Y}}_{ij}=Y_{ij}-f_{j}({\bar{Y}}_{i})={\bar{Y}}_{i}+E_{ij}.$$

**Figure 2:**
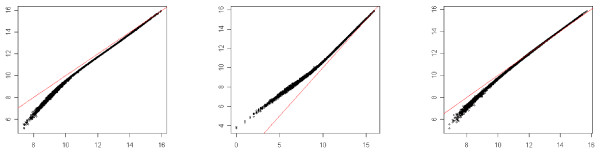
**Normalization function**. *x*-axis: raw data for one channel, *y*-axis: normalized data from the same channel. First column: *Cy*5, second column: *Cy*3, third column: *Alexa*594.

**Figure 3:**
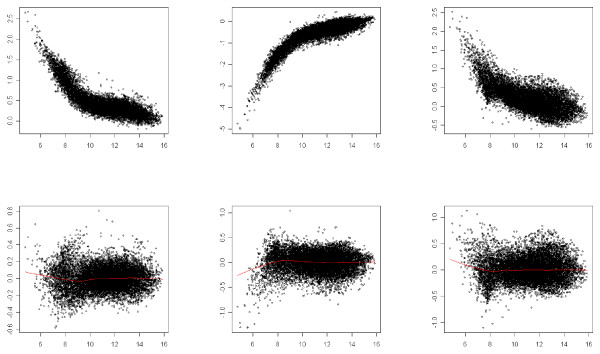
**Modified-MA-plots**. *x*-axis: mean intensity, *y*-axis: difference between channel and mean intensities. First row: raw data, last row: normalized data. First column: *Cy*5, second column: *Cy*3, third column: *Alexa*594.

**Figure 4:**
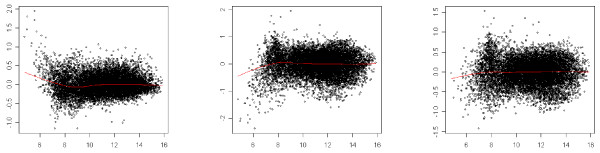
**Usual MA-plots**. *x*-axis: mean intensity between two channels, *y*-axis: difference between two channels. First column: *Cy*5 – *Cy*3, second column: *Cy*3 – *Alexa*594, third column: *Cy*5 – *Alexa*594.

We point out that if this normalization procedure is applied on a two-color microarray, it leads back to the usual lowess method. Figures 2, 3 and 4 illustrate the result of the normalization procedure on an array issued from the Forster triple-self dataset. Figure 2 contains the plots showing the normalization function for each channel. In the context of two-color microarray, the MA-plot is the main graphical representation for visualizing the effect of the global dye-bias normalization. Figure 3 contains the modified MA-plots for three dyes. In such plots, the *x*-axis coordinate is the mean intensity of the three channels ${\bar{Y}}_{i}$ and the *y*-axis coordinate is the difference between intensity of channel *j *and the mean intensity, $D_{ij}=Y_{ij}-{\bar{Y}}_{i}$. Figure 3 contains the similar modified-MA-plots for the normalized data. The three usual MA-plot of the normalized data for each couple of dyes are represented in Figure 4.

### Validation of the normalization

The normalization procedure has to be validated on two points: first it must suppress or at least cut the technical bias and second it must not reduce the difference of expression between genes. We have used different experiments to check both points. We first use an analysis of variance (Anova) approach, and then a count of the number of differentially expressed genes.

#### Analysis of variance of raw and normalized data

Kerr et al. [4] proposed to validate a given normalization method by analyzing the raw and the normalized data through the same Anova model. A good normalization method should cut the sum of squares due to technical factors or interactions and should not decrease the sum of squares due to the interesting biological term under consideration, the gene-condition interaction. As expected, the normalization reduces all the technical biases and the gene-condition interaction is only slightly decreased. This proves that the normalization is effective (see Table 4).

**Table 4:** Anova Sum of Squares before and after normalization (URGV3 data set)

Source	Before normalization	After normalization
Array	1191	1184
Dye	13269	11
Array*Dye	425	43
Gene	310836	309177
Array*Gene	6362	6378
Dye*Gene	10595	2739
Condition*Gene	2387	2105
Residual	24890	23929

#### Number of genes declared differentially expressed

One way for checking the effciency of a normalization method is to analyze self-experiments, where only one sample is labeled with all the dyes and then hybridized on the same array. In such experiments, no differentially expressed gene is expected. Differential analysis with *varmixt *([1]) of the triple-self arrays of Forster's experiment and of the URGV2 dataset gives no genes differentially expressed after normalization. A good normalization procedure should not decrease the true difference of expression between genes. We have compared the number of differentially expressed genes for two microarray experiments, studying three conditions:

1. 3 triple-target microarrays (see URGV3 in the Methods Section)

2. 6 two-color microarrays (see URGV4 in the Methods Section), a dye-swap for each comparison between two of the three conditions.

Table 5 states the number of differentially expressed genes for each comparison and for each experiment. The two-color microarrays have been normalized using the usual lowess method and the triple-target microarrays have been normalized by equation (1). All other steps of normalization and the statistical method for differential analysis are the same for the two experiments. The experiment with three triple-target microarrays gives more differentially expressed genes than the experiment with six two-color microarrays, which proves that the proposed normalization for triple-target microarrays does not reduce the true difference between gene expression more than the usual lowess method for two dyes does.

**Table 5:** Number of genes declared differentially expressed for triple-target and two-color experiments Number of differentially expressed genes (FDR = 5%).

Comparison	Triple-target experiments	Two-color experiments	Common
C1 versus C2	3353	2188	1925
C1 versus C3	3986	3384	2737
C2 versus C3	4519	3465	2928

## Conclusion

The proposed normalization procedure is effective: the number of false positives is under control, and the triple-target microarray experiments are more powerful than the corresponding two-color experiments.

There is thus room for improving the routine two-color microarray experiments. The normalization procedure proposed could be used for any number of channels *p *> 2, so that it could be tested for four-target microarrays or used to evaluate the bleeding of *Alexa *488.

## Methods

### Correction of bleeding

As the bleeding seems to work on a linear scale, a natural idea is to estimate *p*(*p *- 1) bleeding coefficients and correct the raw data using the following expression:

(2) $${\tilde{X}}_{ij}=X_{ij}-\sum_{l\neq j}\beta_{lj}Xil$$

where *X*_*ij *_is the raw measure of expression of gene *i *on channel *j*, ${\tilde{X}}_{ij}$ is the corresponding value corrected for bleeding, and *β*_*lj *_is the coefficient of bleeding from channel *l *to channel *j*. This bleeding correction works under two assumptions:

1. the bleeding coefficients do not depend on the intensity of the bleeding channel,

2. the effects of the bleeding from several channels are additive on a linear scale.

The first assumption is confirmed by the preceding analysis (see Results Section) and the second one seems realistic. Two ways for estimating the coefficients *β*_*lj *_are possible:

1. use preliminary experiment with *p *slides single-target hybridization,

2. use the current data set, with all the *p*-target hybridization slides.

The model framework for estimating the bleeding coefficients in *p*-target experiments is the following:

(3) $$X_{ija}=\mu +\alpha_{i}+\gamma_{j}+\eta_{ij}+\xi_{a}+\tau_{ja}+\delta_{c(j,a)}+\theta_{ic(j,a)}+\sum_{l\neq j}\beta_{lja}X_{ila}+E_{ija}$$

where *a *is the array index, *X*_*ija *_is the measure of expression for gene *i*, channel *j *and array *a*, *c*(*j*, *a*) is the condition associated with channel *j *and slide *a*, *α*_*i *_is the gene effect, *γ*_*j *_the dye effect, *η*_*ij *_the interaction between gene *i *and dye *j*, *ζ*_*a *_the effect of array *a*, *τ*_*ja *_the interaction between dye *j *and array *a*, *δ*_*c*_(*j*, *a*) the condition *c*(*j*, *a*) effect, *θ*_*ic*_(*j*, *a*) the interaction gene-condition and *β*_*lja *_is the bleeding coefficient from channel *l *to channel *j *for array *a*. Note that the global condition effect *δ*_*c*_(*j*, *a*) is included in the interaction *τ*_*ja*_. This is a standard linear model. However the size of the design matrix is huge (more than 2*np*) so the computation is not routinely feasible. Even if the computation were feasible, simulations show that there are many confounding effects in this statistical model and consequently the estimates of the *β*_*lja *_are not reliable (data not shown).

Therefore the only possible procedure is to estimate the bleeding coefficients on preliminary one-target slides. This procedure assumes that the coefficients do not depend on the microarray and that the bleeding coefficients of the preliminary single-target experiments are the same as in real *p*-target experiments. The bleeding effect may depend on the platform and the technology (laser, PMT tuning, image analysis). This implies that the machine-tuning parameters are not modified during the experiment. For the bleeding correction of the URGV data sets we have used Equation (2) with the coefficients of Table 2. In practice we have only corrected the bleeding from *Cy*5 to *Alexa*594, from *Cy*3 to *Alexa*594 and from *Alexa*594 to *Cy*3.

### Labelling and hybridization protocols for microarray experiments

Microarray analysis was carried out at the Unité de Recherche en Génomique Végétale (Evry, France), using the CATMA array (Crowe et al., 2003; Hilson et al., 2004), containing 24 576 gene-specific tags from *Arabidopsis thaliana*. Total RNA was extracted from each sample using TRIzol extraction (Invitrogen, Carlsbad, CA) followed by two ethanol precipitations, then checked for RNA integrity with the Bioanalyzer from Agilent (Waldbroon, Germany). cRNAs were produced from 1 *μ*g of total RNA from each sample with the "Message Amp aRNA" kit (Ambion, Austin, TX). Then 5 *μ*g of cRNAs were reverse transcribed in the presence of 200 u of SuperScript II (Invitrogen, Carlsbad, CA), in presence of Amino-allyl-dUTP (Sigma-Aldrich, St. Louis, MO). The samples are then labelled by coupling with Cy3 or Cy5 monoreactive dyes (G.E. Healthcare, UK) or Alexa Fluor 594 (Invitrogen, Carlsbad, CA). Labelled samples were purified and concentrated with Qiaquick columns (Qiagen, Hilden, Germany). Slides were pre-hybridized for 1 h and hybridized overnight at 42°C in 25% formamide. Slides were washed in 2 × SSC+ 0.1% SDS 4', 1 × SSC 4', 0.2× SSC 4', 0.05 × SSC1' and dried by centrifugation. The slides were scanned on a Genepix Professionnal 4200A scanner (Molecular Devices Corporation, St. Grégoire, France) and images were analysed by Genepix Pro 6.0 (Molecular Devices, St. Grégoire, France).

### URGV Dataset description

#### URGV1 single target hybridization microarray experiment

Total RNA sample from Arabidopsis thaliana flowers was reverse-transcribed and labelled in a one-dye fashion either with cy3, cy5 or Alexa Fluor 594 and hybridized separately on two slides each (i.e. six hybridizations).

#### URGV2 triple-self hybridization microarray experiment

One pool of total RNA from Arabidopsis thaliana roots, leaves and flowers was separated in three aliquots and reverse-transcribed and labelled with the three fluorochromes, then melted and hybridized on the same slides in three technical replicates (i.e. three hybridizations).

#### URGV3 Triple target experiment

Total RNA from Arabidopsis thaliana roots, leaves and flowers were labelled independently with the three fluorochromes in a one-dye fashion either with cy3, cy5 or Alexa Fluor 594. Then the three samples were hybridized on the same slide, each being labelled with a different fluorochrome, in three technical replicates with fluorochrome switch (i.e. three hybridizations).

#### URGV4 dual target experiment

Total RNA from Arabidopsis thaliana roots; leaves and flowers were labelled independently with the three fluorochromes in a one-dye fashion either with cy3, cy5 or Alexa 594. Then two samples were hybridized on the same slide, each being labelled with a different fluorochrome. Each comparison was performed with a technical replicate with fluorochrome switch: regular dye-swap (i.e. six hybridizations).

## Authors' contributions

MLMM, JA, ABH and JJD designed the method. MLMM, JA and JJD wrote the manuscript. JA implemented part of the software and performed the statistical analysis. SE made the URGV experiments under the direction of JPR. FM implemented part of the software. All authors contributed to the discussion and have approved the final manuscript.
